# Supplementary figures and images for: Insight into bacterial and archaeal community structure of Suaeda altissima and Suaeda dendroides rhizosphere in response to different salinity level
Source: Microbiol Spectr. 2023 Dec 1;12(1):e01649-23. doi: 10.1128/spectrum.01649-23 (PMC10783136; doi:10.1128/spectrum.01649-23)

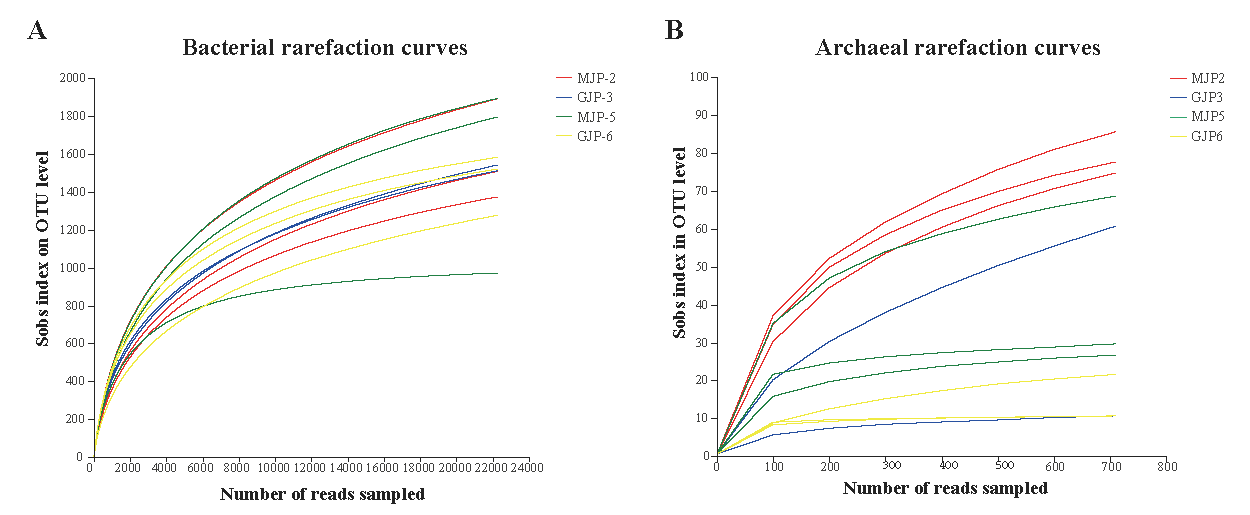

Supplement: Fig. S1 — Rarefaction curves based on the sequences of the V4 region of the 16S rRNA gene from samples associated with rhizosphere soil samples from Suaeda dendroides and Suaeda altissima. [file spectrum.01649-23-s0001.tif]

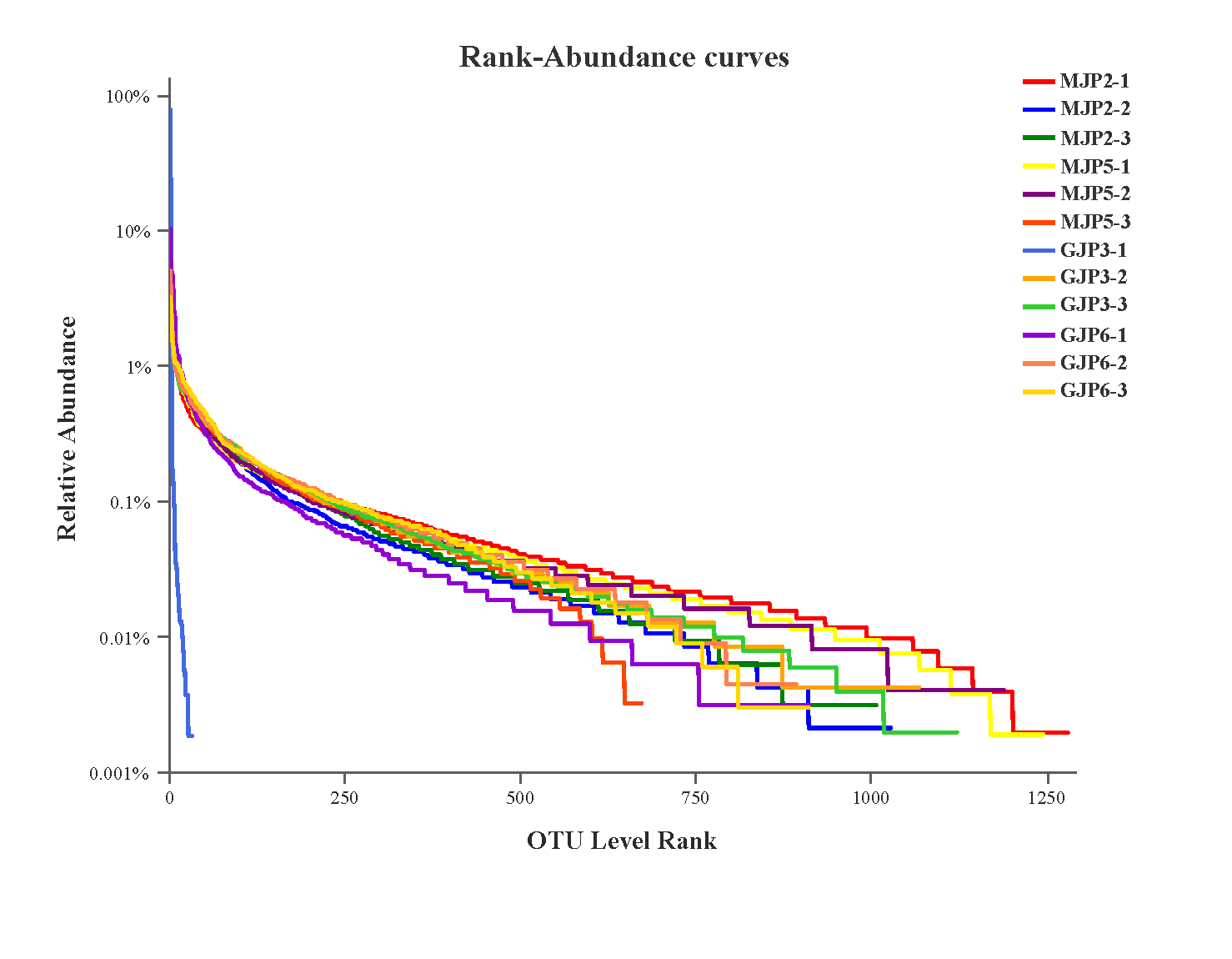

Supplement: Fig. S2 — Rank-abundance curves on OTU level from samples associated with rhizosphere soil samples from Suaeda dendroides and Suaeda altissima. [file spectrum.01649-23-s0002.tif]
