# Supplementary material for: Insight into bacterial and archaeal community structure of Suaeda altissima and Suaeda dendroides rhizosphere in response to different salinity level
Source: Microbiol Spectr. 2023 Dec 1;12(1):e01649-23. doi: 10.1128/spectrum.01649-23 (PMC10783136; doi:10.1128/spectrum.01649-23)
Supplement: Supplemental legends — Legends for supplemental tables and figures. [file spectrum.01649-23-s0003.docx]

**Supplement Table Legends**

**Table S1** The classes of soil salinity and the growth status of crop.

**Table S2** The information of sampling location

**Table S3** Adonis analysis were used for comparing the differences between groups.

**Table S4A** the correlation analysis between alpha diversity index of bacteria and soil properties by Pearson correlation test.

**Table S4B** the correlation analysis between alpha diversity index of archaea and soil properties by Pearson correlation test.

**Table S5A** Node properties and centrality coefficients of the bacterial covariance correlation network in MJP-2 sample.

**Table S5B** Node properties and centrality coefficients of the bacterial covariance correlation network in GJP-3 sample.

**Table S5C** Node properties and centrality coefficients of the bacterial covariance correlation network in MJP-5 sample.

**Table S5D** Node properties and centrality coefficients of the bacterial covariance correlation network in GJP-6 sample.

**Table S5E** Node properties and centrality coefficients of the archaeal covariance correlation network in MJP2 sample.

**Table S5F** Node properties and centrality coefficients of the archaeal covariance correlation network in GJP-3 sample.

**Table S5G** Node properties and centrality coefficients of the archaeal covariance correlation network in MJP-5 sample.

**Table S5H** Node properties and centrality coefficients of the archaeal covariance correlation network in GJP-6 sample.

**Table S6A** Node properties and center coefficients of bacterial co-occuriance networks in all samples.

**Table S6B** Node properties and center coefficients of archaeal co-occuriance networks in all samples.

**Supplement Figure legends**

**FIG S1** Rarefaction curves based on the sequences of the V4 region of the 16S rRNA gene from samples associated with rhizosphere soil samples from *Suaeda dendroides* and *Suaeda altissima*.

**FIG S2** Rank-abundance curves on OTU level from samples associated with rhizosphere soil samples from *Suaeda dendroides* and *Suaeda altissima*.
