# Supplementary material for: Insight into bacterial and archaeal community structure of Suaeda altissima and Suaeda dendroides rhizosphere in response to different salinity level
Source: Microbiol Spectr. 2023 Dec 1;12(1):e01649-23. doi: 10.1128/spectrum.01649-23 (PMC10783136; doi:10.1128/spectrum.01649-23)
Supplement: Supplemental tables — Tables S1 to S4. [file spectrum.01649-23-s0004.docx]

| **Table S1** The classes of soil salinity and the growth status of crop. | | |
| --- | --- | --- |
| Soil salinity class | Conductivity of the soil saturation extract (dS m^-1^) | Effect on crop plants |
| Nonsaline | 0-2 | Saline effects negligible |
| Slightly saline | 2-4 | Yields of sensitive crops may be restricted |
| Moderately saline | 4-8 | Yields of many crops are restricted |
| Strongly saline | 8-16 | Only tolerant crops yield satisfactorily |
| Very strongly saline | >16 | Only a few very tolerant crops yield satisfactorily |

| **Table S2** The information of sampling location | | | | | | |
| --- | --- | --- | --- | --- | --- | --- |
| Location | Sampling location | Sample coordinates | Plant  species | Subsamples | EC_25_  (mS/cm） | Soil salinity class |
| A  （MJP-2） | Jiahezi Reservoir | N：44°27′25″  E:86°7′38″ | *Suaeda dendroides* | MJP2-1 | 14.19 | Strongly saline |
|  |  |  |  | MJP2-2 |  |  |
|  |  |  |  | MJP2-3 |  |  |
| B  （GJP-3） | Murshroom Lack | N：44.417253°  E：85.938723° | *Suaeda altissima* | GJP3-1 | 13.15 | Strongly saline |
|  |  |  |  | GJP3-2 |  |  |
|  |  |  |  | GJP3-3 |  |  |
| C  （MJP-5） | An Jihai | N：44.389127°  E：85.494197° | *Suaeda dendroides* | MJP5-1 | 17.05 | Very strongly saline |
|  |  |  |  | MJP5-2 |  |  |
|  |  |  |  | MJP5-3 |  |  |
| D  （GJP-6） | Shihezi Fourth battalions and sixteenth companies | N：44°25′30″  E：85°31′39″ | *Suaeda altissima* | GJP6-1 | 16.57 | Very strongly saline |
|  |  |  |  | GJP6-2 |  |  |
|  |  |  |  | GJP6-3 |  |  |

| **Table S3** Adonis analysis were used for comparing the differences between groups. | | | | | | | |
| --- | --- | --- | --- | --- | --- | --- | --- |
|  |  | Df | Sums of Sqs | Mean Sqs | F. Model | R^2^ | Pr(>F) |
| Bacteria | group factor | 3 | 0.9699 | 0.3233 | 1.9351 | 0.4534 | 0.045 |
|  | Residuals | 7 | 1.1695 | 0.1671 | - | 0.5466 | - |
|  | Total | 10 | 2.1394 | - | - | 1.0000 | - |
| Archaea | group factor | 3 | 0.9170 | 0.3057 | 1.6500 | 0.4142 | 0.098 |
|  | Residuals | 7 | 1.2968 | 0.1853 | - | 0.5858 | - |
|  | Total | 10 | 2.2139 | - | - | 1 | - |

| **Table S4A** the correlation analysis between alpha diversity index of bacteria and soil properties by Pearson correlation test. | | | | | | | | | | | |
| --- | --- | --- | --- | --- | --- | --- | --- | --- | --- | --- | --- |
| Index | Shannon |  | Simpson |  | Ace |  | Chao1 |  | Sobs |  |  |
|  | r | Sig. | r | Sig. | r | Sig. | r | Sig. | r | Sig. |  |
| OM | 0.122 | 0.706 | -0.070 | 0.829 | -0.166 | 0.607 | -0.180 | 0.575 | -0.107 | 0.740 |  |
| TN | 0.062 | 0.848 | -0.012 | 0.970 | -0.319 | 0.312 | -0.318 | 0.313 | -0.218 | 0.495 |  |
| TP | -0.015 | 0.963 | 0.027 | 0.933 | -0.294 | 0.353 | -0.269 | 0.398 | -0.291 | 0.359 |  |
| TK | -0.079 | 0.808 | 0.051 | 0.874 | -0.188 | 0.560 | -0.155 | 0.629 | -0.211 | 0.511 |  |
| AHN | 0.067 | 0.836 | -0.034 | 0.916 | -0.332 | 0.292 | -0.324 | 0.304 | -0.276 | 0.386 |  |
| AP | -0.031 | 0.924 | 0.043 | 0.895 | -0.322 | 0.307 | -0.298 | 0.346 | -0.292 | 0.358 |  |
| AK | -0.034 | 0.916 | 0.035 | 0.913 | -0.301 | 0.342 | -0.275 | 0.387 | -0.290 | 0.361 |  |
| pH | -0.108 | 0.738 | 0.079 | 0.808 | 0.311 | 0.326 | 0.311 | 0.326 | 0.221 | 0.489 |  |
| Cl^-^ | -0.053 | 0.869 | -0.009 | 0.977 | 0.280 | 0.378 | 0.272 | 0.392 | 0.227 | 0.479 |  |
| K^+^ | -0.072 | 0.823 | 0.106 | 0.744 | -0.300 | 0.343 | -0.277 | 0.383 | -0.248 | 0.436 |  |
| Mg^2+^ | 0.041 | 0.900 | -0.136 | 0.673 | 0.091 | 0.777 | 0.071 | 0.825 | 0.204 | 0.525 |  |
| Na^+^ | 0.047 | 0.886 | -0.197 | 0.538 | 0.182 | 0.572 | 0.187 | 0.561 | 0.059 | 0.856 |  |
| HCO_3_^-^ | 0.216 | 0.500 | -0.324 | 0.304 | -0.088 | 0.785 | -0.100 | 0.757 | 0.063 | 0.846 |  |
| SO_4_^2-^ | -0.007 | 0.982 | -0.138 | 0.670 | 0.166 | 0.607 | 0.168 | 0.602 | 0.134 | 0.677 |  |
| Ca^2+^ | -0.115 | 0.721 | 0.063 | 0.845 | 0.256 | 0.421 | 0.247 | 0.439 | 0.249 | 0.436 |  |
| EC | 0.021 | 0.947 | -0.114 | 0.725 | -0.096 | 0.767 | -0.060 | 0.853 | -0.212 | 0.509 |  |
| Catalase | 0.124 | 0.701 | -0.130 | 0.686 | 0.169 | 0.600 | 0.141 | 0.662 | 0.181 | 0.574 |  |
| Urease | -0.056 | 0.864 | 0.154 | 0.633 | -0.290 | 0.361 | -0.282 | 0.375 | -0.215 | 0.502 |  |
| Phosphatase | 0.064 | 0.843 | 0.024 | 0.941 | 0.045 | 0.889 | 0.016 | 0.961 | 0.113 | 0.726 |  |
| Sucrase | 0.115 | 0.722 | -0.277 | 0.383 | -0.183 | 0.570 | -0.155 | 0.630 | -0.239 | 0.455 |  |
| Protease | -0.175 | 0.586 | 0.268 | 0.401 | -0.147 | 0.648 | -0.130 | 0.686 | -0.120 | 0.709 |  |
| Note: * indicates that there is a significant correlation between the two variables, and Sig. value <0.05; ** indicates that there is extremely significant correlation between the two variables, Sig. value < 0.01, "-" indicates a negative correlation. CAT, catalase; Ure, urease; PHO, phosphatase; SUR, Sucrase. | | | | | | | | | | | |

| **Table S4B** the correlation analysis between alpha diversity index of archaea and soil properties by Pearson correlation test. | | | | | | | | | | | |
| --- | --- | --- | --- | --- | --- | --- | --- | --- | --- | --- | --- |
| Index | Shannon |  | Simpson |  | Ace |  | Chao1 |  | Sobs |  |  |
|  | r | Sig. | r | Sig. | r | Sig. | r | Sig. | r | Sig. |  |
| OM | 0.083 | 0.797 | -0.346 | 0.270 | -0.590^*^ | 0.044 | -0.599^*^ | 0.040 | 0.048 | 0.883 |  |
| TN | -0.244 | 0.444 | -0.030 | 0.925 | -0.864^**^ | 0.000 | -0.859^**^ | 0.000 | -0.290 | 0.361 |  |
| TP | -0.719^**^ | 0.008 | 0.581^*^ | 0.048 | -0.821^**^ | 0.001 | -0.798^**^ | 0.002 | -0.734^**^ | 0.007 |  |
| TK | -0.715^**^ | 0.009 | 0.729^**^ | 0.007 | -0.416 | 0.179 | -0.392 | 0.207 | -0.705^*^ | 0.010 |  |
| AHN | -0.451 | 0.142 | 0.185 | 0.565 | -0.940^**^ | 0.000 | -0.932^**^ | 0.000 | -0.485 | 0.110 |  |
| AP | -0.693^*^ | 0.013 | 0.551 | 0.064 | -0.812^**^ | 0.001 | -0.791^**^ | 0.002 | -0.711^**^ | 0.010 |  |
| AK | -0.723^**^ | 0.008 | 0.615^*^ | 0.033 | -0.741^**^ | 0.006 | -0.719^**^ | 0.008 | -0.734^**^ | 0.007 |  |
| pH | 0.288 | 0.365 | -0.009 | 0.978 | 0.856^**^ | 0.000 | 0.855^**^ | 0.000 | 0.331 | 0.293 |  |
| Cl^-^ | 0.435 | 0.157 | -0.169 | 0.600 | 0.929^**^ | 0.000 | 0.924^**^ | 0.000 | 0.454 | 0.138 |  |
| K^+^ | -0.575 | 0.050 | 0.464 | 0.129 | -0.707^*^ | 0.010 | -0.682^*^ | 0.015 | -0.601^*^ | 0.039 |  |
| Mg^2+^ | 0.556 | 0.061 | -0.424 | 0.170 | 0.680^*^ | 0.015 | 0.669^*^ | 0.017 | 0.505 | 0.094 |  |
| Na^+^ | -0.067 | 0.837 | 0.292 | 0.357 | 0.643^*^ | 0.024 | 0.632^*^ | 0.027 | -0.039 | 0.905 |  |
| HCO_3_^-^ | 0.057 | 0.861 | -0.153 | 0.635 | -0.070 | 0.828 | -0.096 | 0.768 | -0.019 | 0.953 |  |
| SO_4_^2-^ | 0.052 | 0.873 | 0.192 | 0.549 | 0.730^**^ | 0.007 | 0.722^**^ | 0.008 | 0.042 | 0.898 |  |
| Ca^2+^ | 0.480 | 0.114 | -0.206 | 0.520 | 0.932^**^ | 0.000 | 0.929^**^ | 0.000 | 0.470 | 0.123 |  |
| EC | -0.691^*^ | 0.013 | 0.796^**^ | 0.002 | -0.087 | 0.789 | -0.080 | 0.805 | -0.699^*^ | 0.011 |  |
| Catalase | 0.586^*^ | 0.045 | -0.608^*^ | 0.036 | 0.368 | 0.239 | 0.343 | 0.275 | 0.578^*^ | 0.049 |  |
| Urease | -0.286 | 0.368 | 0.073 | 0.823 | -0.812^**^ | 0.001 | -0.794^**^ | 0.002 | -0.314 | 0.321 |  |
| Phosphatase | 0.622^*^ | 0.031 | -0.767^**^ | 0.004 | -0.016 | 0.961 | -0.031 | 0.925 | 0.598^*^ | 0.040 |  |
| Sucrase | -0.859^**^ | 0.000 | 0.864^**^ | 0.000 | -0.350 | 0.265 | -0.349 | 0.266 | -0.858^**^ | 0.000 |  |
| Protease | -0.270 | 0.397 | 0.243 | 0.446 | -0.379 | 0.225 | -0.352 | 0.262 | -0.269 | 0.399 |  |
| Note: * indicates that there is a significant correlation between the two variables, and Sig. value <0.05; ** indicates that there is extremely significant correlation between the two variables, Sig. value < 0.01, "-" indicates a negative correlation. CAT, catalase; Ure, urease; PHO, phosphatase; SUR, Sucrase. | | | | | | | | | | | |
